# Supplementary material for: Functional Similarity and Difference among Bra-MIR319 Family in Plant Development
Source: Genes (Basel). 2019 Nov 21;10(12):952. doi: 10.3390/genes10120952 (PMC6947622; doi:10.3390/genes10120952)
Supplement: Supplementary file 1 [file genes-10-00952-s001.pdf]

Supplementary Figure 1

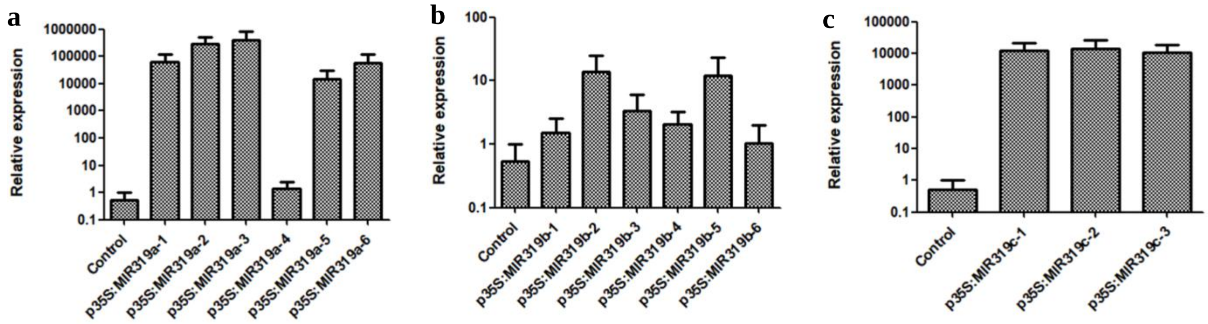

Figure 1. Expression analysis of *Bra-MIR319a*. (**a**), *Bra-MIR319b* (**b**), and *Bra-MIR319c* (**c**) in transgenic plants by qRT-PCR. Table S1: Primers used in this article.

Supplementary Table 1

Primers

**Gene name**	**Forward primer sequence (5'-3')**	**Reverse primer sequence (5'-3')**
Bra-miR319	TTGGACTGAAGGGAGCTCCCT	
BcU6	CCGATAAAATTGGAACGATACAG	ATTTCTCGATTTGTGCGTGTC
Bra-MIR319a	TTATTAGTTGGTATTGTAGGGA	GAAAATTATAGTAGTAAGAGAGAGG
Bra-MIR319b	CTCATTCATCCAAATACCAA	CCCTTCAGTCCAAGCATAG
Bra-MIR319c	TGAAGCGAGACTGAAATAGAT	ACACAGATAAAGAGAGATGAA
BcUBC10	GGGTCCTACAGACAGTCCTTAC	ATGGAACACCTTCGTCCTAAA
BcMYB101-1	ATGCACGGTGGTGGAGAGAC	TCAGTAACATGACCCCAACAAG
mBcMYB101-1	ATGCACGGTGGTGGAGAGAC	TAACTGCGAAAATGGGTCGTTGTTAG<br>GGCTAAACGAAGCTCTG
mBcMYB101-1	AGGACTAGACAATGCTGTCCCAGA<br>GCTTCGTTTAGCCCTA	TCAGTAACATGACCCCAACAAG
5' modified<br>RACE	GATGATTGATGAGAAGAGTTTAGA	CTTCACTATCGTTGGCTC ATTGTA
